# Choosing the Right Citation Management Tool: Endnote, Mendeley, Refworks, or Zotero

**DOI:** 10.5195/jmla.2018.468

**Published:** 2018-07-01

**Authors:** Camille Ivey, Janet Crum

**Affiliations:** Library Liaison for Health Sciences, Biomedical Library, Vanderbilt University, Nashville, TN; Head, Content, Discovery, and Delivery Services, Cline Library, Northern Arizona University, Flagstaff, AZ

## INTRODUCTION

Citation management has not always been as easy as it is today. Years ago, references were manually organized on index cards, an awkward and time-consuming process. Citation management software was introduced in the 1980s and used primarily to organize references, search databases for articles on a particular topic, and generate bibliographies [1]. Over the years, users’ needs have changed, technology has advanced, and many new features have been added, including options for social networking and portable document format file (PDF) management.

There are now many bibliographic management packages available and many factors to consider when choosing the product that best meets the needs of the individual user or institution. Popular tools include RefWorks, EndNote, Zotero, Mendeley, and F1000 Workspace. This review will cover the first four; F1000 Workspace was reviewed in the *Journal of the Medical Library Association (JMLA)* in 2017 [2].

First released in 1988 [3], EndNote is a commercial product that is primarily marketed via sales of its desktop application (currently version X8). A basic online version is free, but it has limited features and functionality. RefWorks, first released in 2001 [4], is an entirely web-based application marketed to libraries as an institution-wide tool, though a vendor representative indicated that individual accounts used to be available and will be offered again [5]. The product is currently transitioning to a new interface, referred to by the vendor as “new RefWorks.” Zotero’s free, open source citation manager was initially introduced in 2006 as an extension for the Firefox web browser. It is now available as a standalone application [6]. First released in 2008 [7], Mendeley is a free cloud-based citation manager with desktop and online versions. It also serves as an academic research network, offering a variety of social networking features.

All four products share a core set of features that allow users to import, organize, and manage citations and associated full text. Users can import references from a variety of databases, create in-text citations and bibliographies, and import bibliographic information from web pages. All offer an extensive list of citation styles and the ability to edit existing styles and create new ones.

The remainder of this review focuses on how these products differ with respect to the most commonly used features of citation managers and the advantages and disadvantages of each product. Table 1 summarizes key differences between the products. This review is based primarily on current desktop versions (if applicable) of the products as of February 2018, though online versions are discussed as needed to provide a complete picture of a tool’s functionality. For Mendeley, this review covers the free version only. For RefWorks, this review covers the new RefWorks only; it does not address the older version, known as Legacy RefWorks.

**Table 1:** Citation management tools at a glance

	EndNote	Mendeley	RefWorks	Zotero
Platforms	Mac, Windows	Mac, Windows, Linux	Not applicable (web-based only)	Mac, Windows, Linux
Browsers	Internet Explorer (IE), Firefox, Chrome, Safari	IE, Firefox, Chrome, Safari	IE, Microsoft Edge, Firefox, Safari, Chrome	Firefox, Chrome, Safari
Browser plug-ins	IE (Windows only) and Firefox (Windows and Mac)	IE, Firefox, Chrome, Safari	IE (Windows only), Safari (Mac only), Firefox, Chrome, and Microsoft Edge	Firefox, Chrome, and Safari
Mobile apps	iOS (iPad only)	Android, iOS	None; mobile-friendly site available	None; mobile-friendly site available
Word processing integration	Microsoft Word (Windows and Mac)	Microsoft Word (Windows and Mac), LibreOffice (Linux, Mac, and Windows)	Microsoft Word (Windows and Mac), Google Docs	Microsoft Word (Windows and Mac), Libre Office (Linux, Mac, and Windows)
Importing references	Refer/BibIX, tab delimited, RIS, ISI-CE, filters for hundreds of databases	BibTeX, EndNote, XML, RIS, Zotero library, txt, Ovid (Medlars reprint), PubMed/MEDLINE (nbib), Mendeley web catalog	Mendeley, RIS, filters for hundreds of databases	Bibliontology RDF, BibTeX browser bookmarks, Citavi 5 XML, CSL JSON, EndNote XML, MAB2, MARC, MARCXML, PubMed/MEDLINE (nbib), MODS, Ovid tagged, Primo normalized XML, PubMed XML, RDF, Refer/BibIX, RefWorks tagged, RIS, Web of Science tagged, XML ContextObject
Add reference by identifier	Available by searching external databases in application	ArXiv ID, DOI, PMID	Not available	ISBN, DOI, PMID
Offline availability	Yes, references and files stored locally	Yes, references and files stored locally	Only with link to Dropbox account	Yes, references and files stored locally

## SYSTEM REQUIREMENTS, BROWSER EXTENSIONS, AND MOBILE APPS

All four products offer plug-ins for Microsoft Word. EndNote, Mendeley, and Zotero offer desktop clients, while RefWorks is entirely web-based. Table 1 shows platforms and browser compatibility. All four products offer a web-based version that works with recent versions of popular browsers. Some tools offer plug-ins for other browsers as well, and all offer browser add-ons (bookmarklets, extensions, etc.) for importing bibliographic information from web pages. The Mendeley browser add-on functions only with the online version of Mendeley; the Zotero add-on requires the desktop version for full functionality; and the EndNote add-on can be used in the desktop and online versions.

Of the four products, only EndNote and Mendeley offer mobile apps. While RefWorks and Zotero do not have mobile apps, they do have mobile-friendly sites.

## SEARCHES FOR AND IMPORTING OF REFERENCES

All four tools allow users to import files of references from databases or other citation management tools. Users can search within databases, mark references to save or export, and select from a variety of options to add references to their preferred citation manager tools. Choosing a direct export option opens any of these tools that are installed on users’ computers, and references can be added with one mouse-click. Each of the products has direct export options for at least one of the following databases: PubMed, Web of Science, Science Direct, EBSCO (CINAHL), and ProQuest (PsycINFO). All four systems allow direct export of records from EBSCO (CINAHL), while EndNote is the only tool that has a direct export option for PubMed.

Users can also use the browser add-ons to automatically import references into their reference collections. The add-ons for Mendeley, RefWorks, and Zotero allow users to import references to their reference collections from multiple databases. Depending on the database, users can select individual references or batches, and the references and associated PDFs are imported. Mendeley and Zotero users can use the browser add-ons to import references from PubMed, Web of Science, and Science Direct. Using Zotero, the reviewers were able to import references from ProQuest (PsycINFO), but the Mendeley browser add-on was not able to recognize the bibliographic metadata in ProQuest (PsycINFO) references. Errors were experienced with both Mendeley and Zotero when we imported references from EBSCO (CINAHL).

EndNote’s Capture Reference bookmarklet has more limited functionality than the browser add-ons for the other three products. When displaying a list of PubMed search results, Capture Reference only imported all references on the page; it did not allow us to select specific references to import. Capture Reference did not work at all for us with a list of results from Google Scholar. The only way to import these results was to open each one and then capture it. It also did not directly capture bibliographic information about web pages as easily as the other add-ons did. When we attempted to import information about a web page using Capture Reference, it created an RIS file that we then had to import into EndNote, whereas the other three add-ons added information about web pages directly.

The tools also offer several other ways to add references. Mendeley users can add references by entering a PubMed ID (PMID), digital object (DOI), or ArXivID. Similarly, Zotero users can add references using the international standard book number (ISBN), DOI, or PMID. In the online version of Mendeley, users can search and import references from Mendeley’s web catalog, a collection of all the references that have been added to the personal libraries of Mendeley users [8]. EndNote and RefWorks also allow users to search databases and library catalogs from within the application and import selected search results. EndNote offers an extensive list of free and commercial databases for searching. As of this writing, the new RefWorks only offers PubMed and the Library of Congress as search options, and, when tested, neither search option was functional. According to the RefWorks lead product manager, institutional account administrators can allow users to search any database that is accessible via the Z39.50 search standard. He also indicated that ProQuest is building application programming interfaces (APIs) to integrate RefWorks with other ProQuest tools such as Summon and Primo, which should increase in-app search options [5].

## CREATION OF BIBLIOGRAPHIES

All four applications allow users to create standalone bibliographies in virtually any word processor, including Google Docs. With EndNote, users can create a standalone bibliography by selecting citations and an output style, and copying and pasting into a word processor document. EndNote also allows users to create a subject bibliography that is based on one or more keywords in users’ citations. Both Mendeley and Zotero allow users to drag references from the desktop client into a word processor, where they will be formatted according to the style that users have selected, the quickest and most user-friendly method of bibliography creation. RefWorks includes a feature that allows users to generate a bibliography from a batch of references in a folder, but that feature did not work when we tested it, leaving no way to generate standalone bibliographies from citations.

More commonly, users create bibliographies from in-text citations in a manuscript. All four tools offer Microsoft Word plug-ins to support this functionality. Table 1 provides details about which tools work with other word processors. In EndNote, the bibliography is automatically generated as the citations are inserted into the document. In Mendeley, RefWorks, and Zotero, inserting a citation and creating a bibliography are separate steps, and at least one citation must be added to the document in order to create a bibliography. All four products made occasional small errors in citations, especially when we cited web pages, but Mendeley performed especially poorly, omitting key information from web page citations, such as date accessed.

## MANAGEMENT AND ANNOTATION OF PORTABLE DOCUMENT FORMAT FILES

Each tool offers different options for adding PDF documents. All four systems allow users to add PDF documents by dragging and dropping them into their reference collections and by attaching them to existing citations. EndNote and Mendeley users can drag and drop PDFs both individually and in folders. RefWorks users can only add PDFs one at a time, while Zotero users can add multiple PDFs at once. Mendeley users can also add PDFs by putting them in a designated folder called a Watch Folder. Mendeley monitors the contents of these folders and automatically adds any PDFs to reference collections.

All four products can generate metadata from PDFs to create a citation record, but they use somewhat different methods to do so. When we tested articles from three different journals, all four products extracted metadata inconsistently and occasionally inaccurately. For example, one product extracted metadata completely for a given article, while another failed to extract key information (e.g. author name, page numbers) from the same PDF, and a third failed to import any metadata from the PDF. All products exhibited these failures, though RefWorks appeared to be the least accurate, with at least one significant error with each of the three PDFs that we tested.

All of the products, other than Zotero, support PDF annotation in the application. Zotero users can open PDFs in the application of their choice, annotate them, and save them back to the Zotero database. An add-on called Zotfile [9] allows users to extract annotations and perform other PDF management tasks.

## INTEGRATION WITH LIBRARY COLLECTIONS

EndNote and Zotero can use an openURL link resolver to help users retrieve full text from a library’s electronic collections. Users can specify the baseURL of their libraries’ link resolver in the product settings, and the products will use metadata from a citation in their libraries to attempt to locate full text for that item. In Zotero, this feature is called Library Lookup. Users click on a reference in their collections, and if full text is found, the PDF file can be easily dragged and dropped into their reference collections. EndNote users can access full-text through their institutions by using the Find Full Text feature. Mendeley used to allow integration with a library’s link resolver but no longer offers this feature [8]. For RefWorks, institutional administrators can configure a link resolver for all users at that institution.

## COLLABORATION AND SOCIAL NETWORKING

According to RefWorks documentation, RefWorks users can only share collections with users at their own institutions [10]. The RefWorks senior product manager indicated, however, that as of fall 2017, RefWorks users can share folders with other RefWorks users across institutions [5]. EndNote X7 and X8 users can share with each other in groups of up to 100 members [11]. Mendeley and Zotero users can create both public and private groups [12, 13], though Mendeley users with a free account can create and own only one private group, and private groups created by free accounts are limited to three members [14]. Mendeley offers additional social networking features in the online version that the other products do not provide. Mendeley users can search for and follow other researchers with similar interests and receive updates on actions and events of researchers they are following via the Mendeley Newsfeed [12].

## OFFLINE AVAILABILITY

EndNote, Mendeley, and Zotero collections and documents are stored locally and, therefore, available offline. RefWorks is a purely cloud-based system, so access to the application itself is not available offline. Users can, however, link a DropBox account to RefWorks to provide offline access to full-text documents in RefWorks [10].

## UNIQUE FEATURES

Of the four products, EndNote is the only one that offers a journal matching feature, known as Manuscript Matcher, to help users find the right journal for their manuscripts. Users of the online version can provide their article titles, abstracts, and references, and EndNote will provide a list of journal recommendations based on its analysis of Web of Science citation data [15]. RefWorks is the only product to offer a plug-in for Google Docs, an especially useful feature at universities where Google tools are used heavily by students. It is also the only fully cloud-based product. While both Mendeley and Zotero are free, Zotero is the only open-source product among the four. Its source code is hosted on GitHub and freely available under an AGPLv3 license [16].

## CONCLUSION

All four of the tools reviewed here are usable for standard reference manager functions, and each has strengths and weaknesses. For example, in our testing, Zotero’s browser add-on was the easiest to use and captured data more accurately than the other add-ons did. EndNote offered the most choices for searching databases within the tool, and Zotero generated the most accurate bibliographies. Each also offers unique features that may be especially valuable to certain populations (e.g., RefWorks’ integration with Google Docs, Mendeley’s social networking functions). Often, though, the best choice for a given purpose may be determined by factors other than the functionality of the applications themselves. These factors include cost, support provided by institutions, research needs, familiarity with a product from previous experience, and accessibility for the research team members. For example, if users are working on a systematic review with authors at several institutions, they will need to choose a tool that is accessible to everyone on the team. Since users are not limited to the citation managers supported by their institutions, information professionals need to be familiar with all popular choices in order to guide and support their users effectively.
